# The Toxicological Profile of Active Pharmaceutical Ingredients–Containing Nanoparticles: Classification, Mechanistic Pathways, and Health Implications

**DOI:** 10.3390/ph18050703

**Published:** 2025-05-09

**Authors:** Muhaimin Muhaimin, Anis Yohana Chaerunisaa, Mayang Kusuma Dewi, Alfi Khatib, Aghnia Hazrina

**Affiliations:** 1 Department of Pharmaceutical Biology, Faculty of Pharmacy, Universitas Padjadjaran, Jl, Raya Jatinangor Km 21.5, Sumedang 45363, West Java, Indonesia; 2 Department of Pharmaceutics and Pharmaceutical Technology, Faculty of Pharmacy, Universitas Padjadjaran, Jl, Raya Jatinangor Km 21.5, Sumedang 45363, West Java, Indonesia; 3 Postdoctoral Fellow, Faculty of Pharmacy, Universitas Padjadjaran, Jl, Raya Jatinangor Km 21.5, Sumedang 45363, West Java, Indonesia; 4 Department of Pharmaceutical Chemistry, Faculty of Pharmacy, International Islamic University Malaysia, Kuantan 25200, Pahang, Malaysia; 5 Undergraduate Study Program of Pharmacy, Faculty of Pharmacy, Universitas Padjadjaran, Jl, Raya Jatinangor Km 21.5, Sumedang 45363, West Java, Indonesia

**Keywords:** inflammation, nanoparticles, oxidative stress, physicochemical properties, toxicity

## Abstract

Nanotechnology is the manipulation of matter on an atomic and molecular scale, producing a lot of new substances with properties that are not necessarily easily expected based on present knowledge. Nanotechnology produces substances with unique properties that can be beneficial or harmful depending on their biocompatibility and distribution. Understanding nanomaterial toxicity is essential to ensure their safe application in biological and environmental applications. This review aims to provide a comprehensive overview of nanoparticle toxicity, focusing on their physicochemical properties, mechanisms of cellular uptake, and potential health risks. Key factors influencing toxicity include particle size, shape, concentration, aspect ratio, crystallinity, surface charge, dissolution, and agglomeration. Nanoparticles can induce oxidative stress and inflammation, contributing to adverse effects when inhaled, ingested, or applied to the skin. However, their toxicity may not be limited to just these pathways, as they can also exhibit other toxic properties, such as activation of the apoptotic pathway and mitochondrial damage. By summarizing the current knowledge on these aspects, this article intends to support the development of nanoparticles in a safer way for future applications.

## 1. Introduction

Nanoparticles are defined as materials with dimensions ranging from 1 to 100 nanometers [1]. They can be categorized into several types, including metal-based, carbon-based, polymeric, and silica-based nanoparticles [2]. Each type exhibits distinct properties that make it suitable for specific applications. For instance, metal-based nanoparticles (NPs) such as gold and silver are widely used in drug delivery and imaging due to their unique optical and electronic properties [3,4]. However, their small size also leads to increased reactivity and potential toxicity compared to bulk materials [5,6].

The rapid progression of nanotechnology has led to an increasing presence of NPs in numerous consumer products and industrial applications, resulting in widespread human exposure through inhalation, ingestion, and dermal absorption [7,8,9]. While nanoparticles offer significant biological and biomedical benefits, such as targeted drug delivery, enhanced imaging, and antimicrobial properties [10,11,12], their small size and high reactivity also raise important toxicological concerns [5,13]. Exposure to nanoparticles has been associated with a range of adverse health effects, including respiratory inflammation, oxidative stress, and neurotoxicity. This has raised concerns about public health [14]. These findings underscore the dual nature of NPs as both therapeutic tools and potential health hazards [13,15,16]. Therefore, a comprehensive understanding of their biological interactions and toxicity pathways is essential for the development of safer nanoparticles and the establishment of robust regulatory frameworks [17].

One of the most prominent aspects that contributes to nanoparticle toxicity is its physicochemical characteristics (e.g., size, shape, surface charge, and chemical composition), which are known to vary considerably in relation to toxicity [18,19]. Due to their increased surface area-to-volume ratio, smaller nanoparticles better permeate and disrupt cellular pathways [20]. In addition, the interactions of nanoparticles with biological membranes are greatly influenced by the surface charge. Positively charged nanoparticles tend to exhibit higher toxicity compared to negatively charged ones due to their stronger electrostatic interactions with the negatively charged components of cell membranes, such as phospholipid head groups and membrane proteins [21]. These interactions can lead to increased cellular uptake through endocytosis, disruption of membrane integrity, and induction of oxidative stress, ultimately resulting in enhanced cytotoxic effects [22]. Therefore, surface charge is a critical factor influencing nanoparticle-cell interactions and their associated biological responses [23].

Nanoparticles can induce toxicity via multiple systems. An important mechanism involved in this process is the formation of ROS, which can lead to oxidative stress and therefore cellular damage [24]. Moreover, the direct interactions of nanoparticles with biological molecules can inhibit mitochondrial functions, trigger inflammatory responses, and cause DNA damage [25,26]. It is necessary to have a complete understanding of how NPs influence a wide variety of biological systems due to the intricacy of these interactions [27,28,29].

The findings of research indicate that exposure to NP can have a negative impact on a number of organ systems [30]. The inhalation of nanoparticles based on metals has been linked to a variety of respiratory problems, including inflammation and fibrosis [31]. Research indicates that neuropeptides may have a role in neurodegenerative illnesses by promoting neuroinflammation and apoptosis within the nervous system [32]. Moreover, data indicate that NPs might influence reproductive health by altering endocrine functioning [32,33].

It requires rigorous techniques to assess the toxicity of nanoparticles [34]. Traditional models, such as cell cultures and animal models, have been used, but new technologies such as three-dimensional organoid models are being increasingly preferred to better recapitulate human tissue [34,35]. These advanced models allow scientists to assess the potential toxicity of nanoparticles in a more physiologically relevant environment. In addition, metabolomics has emerged as a tool for evaluating NP-induced metabolic changes that occur at the cellular level [36,37].

As the application of nanoparticles proliferates in various industries, regulatory frameworks must adapt to mitigate possible health hazards. Existing rules differ markedly among countries and frequently lack explicit directives for the safety evaluation of nanoparticles [38,39]. Regulatory organizations must provide comprehensive rules that integrate data from toxicological research to safeguard public safety and promote innovation in nanotechnology [40].

Although nanoparticles provide significant promise for technological and medical advancements, their related health hazards must not be disregarded [41]. Continued study is crucial to elucidate the intricacies of NP toxicity and devise solutions for risk mitigation [31]. The researchers are able to promote safer utilization of nanoparticles across a variety of domains by first gaining an understanding of the physicochemical characteristics that influence toxicity and then employing advanced assessment methodologies [18]. The fact that nanoparticles may be both useful instruments and potential dangers highlights the need to maintain a high level of monitoring throughout the process of these particles’ production and management [42].

The rising use of NPs in a variety of sectors, notably in the realms of biomedicine and technology, has resulted in major concerns over the possible dangers that these particles may pose to human physical health [15]. The objective of this research is to provide a comprehensive overview of the existing information concerning the toxicity of nanoparticles, focusing on their physicochemical features, mechanisms of action, and consequences for public health.

## 2. Types of Toxic Nanoparticles

NPs are being employed in a variety of disciplines, such as medicine, electronics, and environmental applications. Nevertheless, their distinctive characteristics also raise concerns regarding their potential toxicity. This investigation categorizes nanoparticles based on their composition and evaluates their associated toxicological effects (Table 1).

### 2.1. Metal-Based Nanoparticles

Metal-based nanoparticles comprise metals such as silver, gold, copper, and titanium dioxide. They are recognized for their antibacterial qualities and are extensively utilized in medical devices and coatings [48]. These nanoparticles can elicit cytotoxicity by producing reactive oxygen species (ROS) that result in oxidative stress and DNA damage [49]. Silver nanoparticles exhibit more toxicity to human lung cells than bigger particles, attributable to their elevated surface area-to-volume ratio [50].

### 2.2. Carbon-Based Nanoparticles

Carbon-based nanoparticles include substances like carbon nanotubes and graphene [51]. They are esteemed for their robustness and electrical conductivity [15]. Studies demonstrate that inhalation of carbon nanotubes can lead to neurotoxicity and lung inflammation. The dimensions and morphology of these nanoparticles substantially affect their toxicological characteristics [52].

### 2.3. Lipid-Based Nanoparticles

Lipid-based nanoparticles (LNPs) have gained substantial attention in recent years, particularly in the field of drug and vaccine delivery, including mRNA vaccines [53]. These nanoparticles are typically composed of biocompatible lipids such as phospholipids, cholesterol, and ionizable lipids, which enhance encapsulation and cellular uptake of active pharmaceutical ingredients [54]. While generally considered safe, their toxicity can arise from several factors, including lipid composition, particle size, and the presence of PEGylated lipids or surfactants [55]. Studies have reported potential adverse effects such as hepatotoxicity, immune stimulation, and complement activation-related pseudoallergy (CARPA) [56]. The use of ionizable lipids, though beneficial for endosomal escape, can contribute to dose-dependent cytotoxicity. Therefore, optimization of their physicochemical properties is essential to improve safety profiles while maintaining therapeutic efficacy [57].

The inclusion of surfactants in lipid-based nanoparticles modifies their cytotoxic profile by influencing surface charge, stability, and biological interactions. Cationic surfactants like Cetyltrimethylammonium Bromide (CTAB) and Didodecyldimethylammonium Bromide (DDAB) increase positive charge, enhancing cellular uptake but also raising cytotoxicity [58,59], while non-ionic surfactants such as Tween 80 and Poloxamers neutralize charge and improve biocompatibility [60,61]. However, certain surfactants like SDS can be inherently cytotoxic, highlighting the importance of careful selection [62].

### 2.4. Protein-Based Nanoparticles

Protein-based nanoparticles are synthesized from natural or recombinant proteins and are utilized in various biological applications [63]. While these nanoparticles typically exhibit lower toxicity compared to synthetic nanoparticles, they may still elicit immunological responses, particularly when subjected to denaturation or aggregation. Such alterations in their structural integrity can trigger immune activation, potentially leading to adverse effects [63,64].

### 2.5. Polymeric Nanoparticles

Polymeric nanoparticles are typically synthesized using biodegradable synthetic polymers such as poly (lactic-co-glycolic acid) (PLGA) or natural polymers like chitosan [65]. Although these carriers are generally considered biocompatible, their degradation products may exert cytotoxic effects, potentially inducing inflammatory responses or other adverse biological effects. The extent of these outcomes is largely influenced by the chemical composition and degradation kinetics of the specific polymer used [65].

### 2.6. Silica Nanoparticles

Silica nanoparticles are widely utilized in drug delivery systems owing to their high surface area and porous architecture, which enable efficient loading and controlled release of therapeutic agents. However, SiNPs have been reported to induce oxidative stress and inflammatory responses in biological systems. The degree of their cytotoxicity is largely dependent on physicochemical properties such as particle size, surface charge, and functionalization, which influence cellular uptake and bioreactivity [66,67,68,69,70].

Table 2 summarizes examples of toxic effects of NPs along with their toxic effects, the organisms or cells tested, concentrations used, exposure conditions, and sources of information. The data highlights the diverse toxicological profiles of these materials based on existing literature.

Organoid models offer significant advantages over traditional animal testing for nanoparticle toxicity assessment [100]. Derived from human stem cells, organoids better replicate the architecture, cellular diversity, and physiological responses of human tissues, thus providing more accurate predictions of human-specific toxicity compared to animal models [100]. They allow for high-throughput screening, can be customized to reflect patient-specific genetics or disease states, and eliminate many ethical concerns associated with animal use [101]. However, traditional animal models still provide critical insights into systemic effects, such as nanoparticle biodistribution, immune system interactions, metabolism, and long-term toxicity, which current organoid systems cannot fully replicate [102]. Animal studies capture the complex interplay between different organs and biological systems under physiological conditions, essential for understanding whole-body responses [103]. Nevertheless, limitations of animal testing include species-specific differences that may limit the translational relevance to humans, ethical issues, and the high costs and extended timeframes associated with in vivo experiments [102]. Thus, while organoid models enhance human relevance and ethical viability in nanoparticle toxicology, traditional animal models remain indispensable for a complete evaluation of systemic toxicity [101,102]^.^

## 3. Mechanisms of NP Toxicity

**Figure 1:**
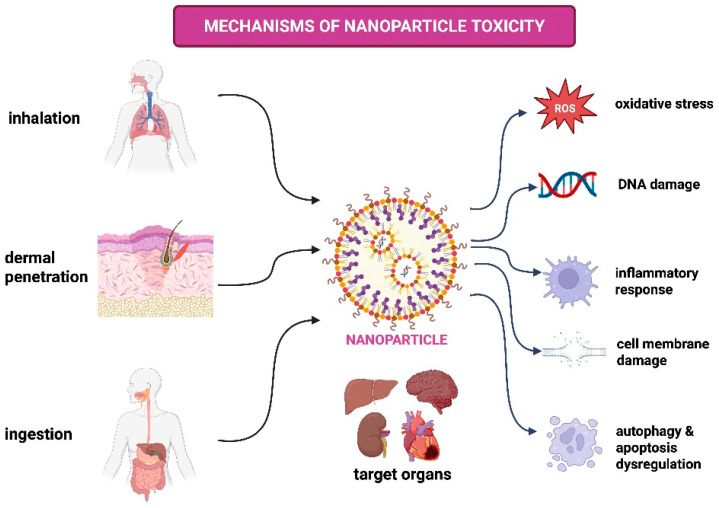
Mechanism of nanoparticle toxicity.

NPs have attracted considerable interest across several domains owing to their distinctive features and potential applications. However, their interaction with biological systems may result in toxicity, necessitating a thorough investigation of the underlying mechanisms [104]. The toxicity associated with nanoparticles, known as nanotoxicity, arises from various physiological reactions initiated by their interactions with biological components [105]. It is imperative to comprehend the manner in which nanoparticles induce hazardous effects in order to assess their safety and effectiveness in applications such as environmental remediation and medication delivery [74]. Figure 1 shows the mechanism of nanoparticle toxicity.

Several strategies can be employed to minimize the generation of reactive oxygen species (ROS) and their associated toxic effects during nanoparticle (NP) synthesis. Surface modification is one effective approach, where nanoparticles are coated with biocompatible materials like polyethylene glycol (PEG) or antioxidants such as ascorbic acid, which shield the nanoparticle surface and reduce their reactivity [106]. Optimizing synthesis parameters, such as temperature, pH, and reaction time, can also help lower ROS production by creating nanoparticles with lower surface energy [107]. Additionally, selecting non-toxic precursors and solvents during synthesis can reduce toxicity and ROS generation [108]. The incorporation of materials with intrinsic antioxidant properties, such as cerium oxide or selenium nanoparticles, can neutralize ROS and reduce oxidative stress [108]. Furthermore, controlling the size and shape of nanoparticles is important, as smaller or sharp-edged nanoparticles tend to produce more ROS; thus, producing spherical nanoparticles can minimize ROS generation [109].

Recent studies have highlighted that the unique physicochemical properties of nanoparticles—particularly size, shape, and surface functionalization—play a crucial role in modulating their toxicological profiles [110]. These characteristics significantly influence the behavior of nanoparticle-based drug delivery systems and must be carefully considered in the development of safe and effective nanopharmaceuticals.

Among these factors, particle size has a particularly significant impact [111]. Smaller nanoparticles, due to their higher surface area-to-volume ratio, tend to exhibit greater reactivity and enhanced membrane permeability, resulting in increased cellular absorption and potential adverse effects [31,74]. Research suggests that nanoparticles with a diameter of approximately 50 nm have the potential to efficiently enter cells and accumulate in various organelles, thereby disrupting the normal functions of cells [112]. This size-dependent reaction has brought to light the need to meticulously design and characterize nanoparticles that are used in biological applications [42]. For instance, the interaction of silver nanoparticles (AgNPs) with biological cells is greatly influenced by their size [110]. Smaller AgNPs, due to their larger surface area relative to volume, tend to be more effective as antibacterial agents [113]. However, this same property also facilitates greater cellular internalization, which can lead to elevated toxicity in mammalian cells through oxidative stress [114]. The morphology of NPs also influences their biological interactions. Various shapes—spherical, rod-shaped, or tubular—affect how particles engage with cell membranes and intracellular structures. Spherical nanoparticles generally exhibit more efficient cellular uptake through endocytosis compared to rod-shaped particles, which tend to induce greater mechanical stress on the cell membrane, potentially leading to membrane disruption and cytotoxicity [115,116]. On the other hand, rod-shaped nanoparticles may induce a stronger inflammatory response due to their larger surface area and longer contact time with cell membranes, which activate immune responses and oxidative stress pathways [115,116]. Studies suggest that elongated nanoparticles may cause higher cellular stress than spherical nanoparticles due to their enhanced surface area and potential for increased mechanical engagement with cellular components [117,118]. Similarly, the shape of silver nanoparticles (AgNPs) significantly affects both their antibacterial efficiency and cytotoxicity. Rod-shaped or sharply edged AgNPs demonstrate enhanced bactericidal activity due to stronger membrane interactions and easier cell penetration [114]. However, these anisotropic shapes also cause higher cytotoxicity in mammalian cells, largely attributed to membrane disruption, oxidative stress, and inflammatory responses [119]. This underscores the importance of thoroughly evaluating nanoparticle morphology to better predict and control their biological behavior.

The interaction between biological systems and nanoparticles is significantly influenced by their surface charge. The stability and interaction of charged nanoparticles with cellular membranes are influenced by their zeta potential, which can result in varying degrees of cytotoxicity [21,120]. Recent studies have demonstrated that the surface charge of nanoparticles (NPs) significantly influences their interactions with cellular membranes and their potential cytotoxicity. Positively charged (cationic) NPs exhibit stronger electrostatic interactions with the negatively charged components of cell membranes, leading to enhanced cellular uptake and increased cytotoxic effects [121]. This interaction can disrupt membrane integrity, resulting in increased permeability, potential membrane destabilization, and cell death [43]. Cationic NPs are also more likely to induce oxidative stress and inflammation, further contributing to their higher toxicity profiles compared to neutral or negatively charged (anionic) NPs [122]. In contrast, anionic NPs tend to have reduced cellular uptake, causing them to exhibit lower cytotoxicity, which can make them more biocompatible for certain applications [123]. Additionally, factors such as nanoparticle size, shape, and surface chemistry also play essential roles in determining the biological interactions and toxicity of NPs [123]. As a result, the surface charge is a crucial factor, but it is not the sole determinant of nanoparticle behavior and toxicity in biological systems [21,27].

The toxicity characteristics of nanoparticles are significantly influenced by their chemical composition [18]. Diverse materials exhibit varying degrees of biocompatibility and reactivity in biological systems [8]. Metal-based nanoparticles, such as silver and gold, have been demonstrated to provoke oxidative stress and inflammation [31,104]. Comprehending the precise interactions between nanoparticle materials and biological molecules is crucial for forecasting toxicological results [74].

The toxic effects of NPs are primarily mediated through mechanisms such as oxidative stress, inflammation, and genotoxicity. Oxidative stress occurs when ROS generation exceeds cellular antioxidant defenses, leading to lipid peroxidation, DNA damage, and apoptosis [25,124]. Concurrently, inflammatory responses triggered by immune activation can result in tissue damage [125,126], while genotoxic effects threaten genetic stability [127].

Recent research highlights that beyond oxidative stress and inflammation, nanoparticles (NPs) can also disrupt cellular homeostasis through mechanisms such as autophagy dysregulation and protein corona formation. NPs can impair the autophagic flux by either blocking autophagosome-lysosome fusion or overwhelming the autophagy machinery, leading to the accumulation of damaged organelles and proteins, which exacerbate cytotoxicity and immune activation [128]. Additionally, when NPs enter biological fluids, they rapidly adsorb proteins onto their surface, forming a “protein corona” that alters their biological identity, cellular uptake, and immunogenicity [129]. This protein corona can modulate NP toxicity by either masking reactive surfaces or, conversely, enhancing recognition by immune cells, leading to exaggerated inflammatory responses or altered biodistribution [130].

**Figure 2:**
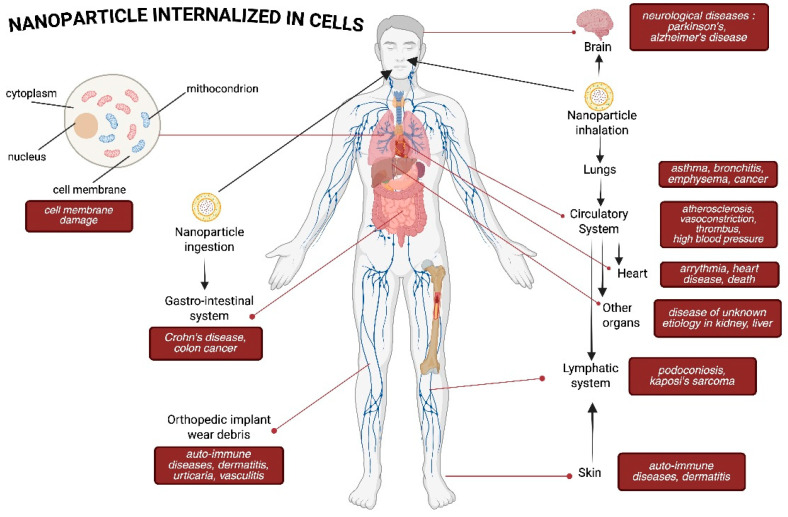
Flowchart mechanism of nanoparticle toxicity in cells.

In conclusion, comprehending the causes of nanoparticle toxicity is essential for guaranteeing safe application across diverse areas (Figure 2). Tailoring NP characteristics—such as size, shape, surface properties, and composition—can mitigate adverse effects and enhance their utility across biomedical and industrial sectors.

## 4. Health Impacts of Nanoparticle Exposure

NPs have attracted considerable interest owing to their extensive use across several sectors and their possible health implications. This study examines the distinct health impacts of NP exposure on several organ systems, namely the respiratory, neurological, and immunological systems.

### 4.1. Respiratory System

The respiratory system serves as a principal entrance channel for inhaled nanoparticles, whose diminutive size enables them to infiltrate the lungs and perhaps access the circulation. Research indicates that exposure to airborne nanoparticles might result in pulmonary inflammation, oxidative stress, and tissue injury [131,132]. For instance, a study involving mice exposed to nano-TiO_2_ at doses of 2.5, 5, and 10 mg/kg body weight for 90 consecutive days showed lung injury, which was associated with alterations in inflammatory-related cytokines and oxidative stress [133]. Chen et al. observed alveolar septal thickening, neutrophil infiltration, and thrombosis in the pulmonary vascular system in mice after an intraperitoneal injection of 324, 648, 972, 1296, 1944 and 2592 mg/kg BW TiO_2_ NPs (3.6 nm) for 7 days, respectively, which demonstrated the generation of inflammation and the blockage of blood vessels in mouse lung [134].

Inhaled nanoparticles may induce both acute and chronic respiratory ailments, including bronchitis, emphysema, and fibrosis [132]. The deposition of nanoparticles in alveolar areas can elicit biological responses from macrophages and dendritic cells, resulting in the secretion of pro-inflammatory cytokines [131,132]. For example, exposure to nickel oxide nanoparticles at a dose of 0.2 mg per rat led to persistent increases in chemokines such as CINC-1 and CINC-2, which are associated with neutrophilic inflammation. [135].

Health Effects:

Acute: Temporary exposure may result in airway irritation, coughing, and respiratory problems [131,136].

Chronic: Long-term inhalation has been linked to conditions such as chronic obstructive pulmonary disease (COPD), asthma exacerbations, and even pulmonary fibrosis due to persistent inflammation and tissue remodeling [131,132].

Example: Titanium dioxide (TiO_2_) and carbon nanotubes have been reported to cause lung inflammation and oxidative damage [131,132].

### 4.2. Nervous System

The nervous system is another critical target for NP toxicity due to the ability of NPs to cross the blood–brain barrier (BBB) and accumulate in neural tissues [137]. NPs can traverse the BBB through several mechanisms, including endocytosis and transcytosis. [138]. These processes allow NPs, such as silver, gold, and silica-based particles, to access sensitive regions of the brain [139]. In addition, there is emerging evidence suggesting that NP exposure may impact neurotransmitter function, alter synaptic plasticity, and influence brain development. The extent of these effects depends on several factors, including NP composition, surface charge, and the duration of exposure [138].

Experimental studies in rodent models, including mice and rats, have demonstrated that exposure to various NPs leads to neurotoxic effects such as oxidative stress, neuroinflammation, and neuronal apoptosis. For instance, Dhakshinamoorthy et al. (2017) reported that mice administered with iron oxide nanoparticles exhibited elevated levels of reactive oxygen species (ROS), increased expression of pro-inflammatory cytokines, and activation of apoptotic markers in brain tissues. These molecular alterations were accompanied by significant behavioral changes, including reduced locomotor activity and impaired spatial memory, as assessed by open field and Morris water maze tests [140].

Similarly, studies on silver nanoparticles have shown that systemic exposure can impair cognitive and motor functions in rodents. Antsiferova et al. (2018) found that prolonged oral administration of silver nanoparticles in mice led to anxiety-like behaviors and deficits in contextual fear conditioning tasks, indicating disruptions in both emotional and memory-related processes [141].

Health Effects:

Neurodegenerative Disorders: Prolonged exposure has been linked to Alzheimer’s and Parkinson’s-like neurodegeneration due to oxidative damage and protein aggregation [142,143,144].

Cognitive Impairments: Studies indicate that some metallic NPs impair learning, memory, and motor coordination [145,146,147].

Example: Silver and zinc oxide NPs have demonstrated neurotoxicity in both in vitro and in vivo models [142,144,147].

### 4.3. Immune System

The immune system is a critical defense mechanism against environmental and pathogenic threats, and its response to NP exposure is of significant concern [148]. NPs have been shown to interact with both innate and adaptive immune cells, resulting in alterations in immune function [149,150]. In the innate immune system, NPs affect cells such as macrophages and dendritic cells, leading to the production of pro-inflammatory cytokines, oxidative stress, and impaired antigen processing [149]. These responses can either enhance pathogen clearance or contribute to chronic inflammation and tissue damage [149].

In contrast, the adaptive immune system is influenced through NP interactions with T cells, either directly or indirectly via altered antigen presentation by dendritic cells [151]. This can result in T-cell activation or suppression, which has been associated with immune dysregulation, including increased susceptibility to infections or the development of autoimmune conditions [21]. Certain nanoparticles, including silica and carbon-based particles, have been shown to enhance the synthesis of autoantibodies and promote immune responses associated with autoimmunity [152].

Moreover, the dual ability of NPs to modulate both NPs and adaptive immunity has made them attractive as vaccine adjuvants [139]. However, understanding the balance between beneficial immune enhancement and harmful immune perturbation is essential to ensure the safety of NP-based therapeutic applications [149].

Specifically, exposure to nanoparticles has been linked to a heightened risk of developing autoimmune disorders [148]. Certain nanoparticles, including silica and carbon-based particles, can induce autoimmunity by modifying the function of antigen-presenting cells and enhancing the synthesis of autoantibodies. Furthermore, NPs can regulate the synthesis of pro-inflammatory cytokines, potentially resulting in persistent inflammation and heightened vulnerability to infections [150].

Health Effects:

Immune Activation: Hyperactivation may lead to persistent inflammation and immunological reactions [150].

Immunosuppression: Suppressed immune function increases vulnerability to infections [153].

Example: Gold NPs have shown immunomodulatory effects, while carbon-based NPs may suppress or overstimulate immune cells [153].

## 5. Conclusions

Nanoparticles present promising applications across medical, industrial, and technological fields; however, their potential health hazards must be carefully considered. As outlined in this review, the toxicity of nanoparticles, particularly metal-, carbon-, lipid-, protein-, polymeric, and silica-based, stems from complex mechanisms such as oxidative stress, inflammation, and cellular damage. These effects can significantly impact the respiratory, neurological, and immune systems. Key physicochemical properties, including size, shape, and surface chemistry, greatly influence nanoparticle interactions with biological systems. Moving forward, more in-depth investigations are needed to understand long-term exposure effects, nanoparticle–biological interactions, and cumulative health impacts. Future research should also emphasize safer nanoparticle design, standardized toxicity assessment methods, and effective regulation. A comprehensive risk assessment and management strategy is essential to minimize health risks and ensure the safe integration of nanotechnology into diverse sectors.

## Figures and Tables

**Table 1:** Overview of nanoparticle types, descriptions, examples, and toxicity effects.

Type of Nanoparticle	Description	Examples	Toxicity Effects	Ref
Metal-Based NPs	Composed of metals or metal oxides, known for their antimicrobial properties.	Silver (Ag), Gold (Au), Copper (Cu), Titanium Dioxide (TiO_2_)	Cytotoxicity, oxidative stress, genotoxicity, and potential organ damage (liver, kidney) due to reactive ions.	[15,43,44]
Carbon-Based NPs	Include carbon nanotubes, graphene, and fullerenes, which are widely used in various applications.	Carbon Nanotubes (CNTs), Graphene, Carbon Black	Neurotoxicity, pulmonary inflammation, and cytotoxicity; size-dependent effects observed in different studies.	[15,45,46]
Lipid-Based NPs	Composed of lipids; often used in drug delivery systems.	Liposomes, Solid Lipid Nanoparticles (SLNs)	Potential for immunotoxicity and cytotoxicity; may induce inflammatory responses depending on lipid composition.	[47]
Protein-Based NPs	Made from proteins; used in drug delivery and vaccine development.	Albumin-based NPs, Silk Fibroin NPs	Generally biocompatible but can induce immune responses; toxicity may arise from protein denaturation or aggregation.	[45,47]
Polymeric NPs	Composed of synthetic or natural polymers, they are versatile in drug delivery applications.	Poly(lactic-co-glycolic acid) (PLGA), Chitosan NPs	Cytotoxicity is related to polymer degradation products; there is potential for inflammatory responses depending on the polymer type.	[15,44]
Silica NPs	Made of silica, and commonly used in biomedical applications and as drug carriers.	Mesoporous Silica NPs	It can induce oxidative stress and inflammation; there is potential for cytotoxic effects depending on particle size and surface modification.	[43,46]

**Table 2:** Toxic Effects of NPs on various organisms and cell types.

Example Nanoparticle	Toxic Effects	Organism/Cell Tested	Concentration	Condition	Ref
Metal-Based					
Silver (AgNPs)	Induces cell death, DNA damage, oxidative stress, and inflammation	Human mesenchymal stem cells (hMSCs), *E. coli*	0.5 to 5 ppm	Various exposure times	[71,72,73]
Gold (AuNPs)	Cytotoxicity, potential genotoxic effects, and inflammation	Human lung adenocarcinoma cells (A-549)	1 to 100 μg/mL	Short-term and long-term exposure	[74,75]
Copper (CuNPs)	Induces oxidative stress, cytotoxicity, and genotoxicity	Various mammalian cell lines	10 to 100 μg/mL	Varies by study	[76,77,78]
Titanium Dioxide (TiO_2_)	Causes oxidative stress, inflammation, and potential lung toxicity	Human lung epithelial cells	0.1 to 10 mg/mL	In vitro exposure	[79,80]
Carbon-Based					
Carbon Nanotubes (CNTs)	Induces oxidative stress, DNA damage, lysosomal damage, mitochondrial dysfunction, and apoptosis	Human lung epithelial cells (A549), macrophages	1 to 100 μg/mL	Various exposure times	[81,82,83,84]
Graphene	Causes oxidative stress, inflammatory responses, and induces TNF-α and IL-6 secretion in macrophages	Human bronchial epithelial cells (BEAS-2B)	1 to 50 μg/mL	In vitro exposure	[85,86,87]
Carbon Black (CB)	Induces pyroptosis, inflammation, and cytotoxicity	THP-1 Monocyte Cells	50–800 μg/mL	In vitro exposure	[88]
Lipid-Based					
Liposomes	Generally low toxicity; potential for hemolysis, cytotoxicity at high concentrations	Human red blood cells, various cancer cell lines	0.1 to 10 mg/mL	In vitro studies	[89,90,91]
Solid Lipid Nanoparticles (SLNs)	Low cytotoxicity; potential for skin irritation, reduced toxicity from essential fatty acids	Various cell lines, human skin fibroblasts	0.1 to 5 mg/mL	In vitro and in vivo studies	[89,90,91]
Protein-Based					
Albumin-based Nanoparticles	Generally low toxicity; minimal immune response; potential for cytotoxicity at high concentrations	Human cancer cell lines, animal models	0.1 to 10 mg/mL	In vitro and in vivo studies	[92,93,94,95]
Silk Fibroin Nanoparticles	Low toxicity; biocompatible; potential for mild inflammatory response in some cases	Human fibroblasts, mouse models	1 to 5 mg/mL	In vitro and in vivo studies	[96,97,98,99]

## Data Availability

Not applicable.
